# Stereotactic Body radiation therapy for liver tumors with or without rotational intensity modulated radiation therapy

**DOI:** 10.1186/1756-0500-6-492

**Published:** 2013-11-27

**Authors:** Elodie Nouhaud, Gilles Créhange, Adèle Cueff, Magali Quivrin, Magali Rouffiac-Thouant, Laurent Mineur, Robin Garcia, Jérôme Chamois, Philippe Maingon

**Affiliations:** 1 Department of Radiation Oncology and Surgical Oncology, Anticancer Centre Georges-François Leclerc, 21079 DIJON CEDEX, France; 2 Biostatistics and epidemiological Unit, EA 4184, Anticancer Centre Georges-François Leclerc, Dijon, France; 3 Department of Radiation Therapy, Sainte-Catherine Institute, Avignon, France

**Keywords:** SBRT, Liver, Radiotherapy, HCC, metastases

## Abstract

**Background:**

To evaluate the feasibility and efficacy of Stereotactic body radiation therapy (SBRT) for primary liver lesions and liver metastases treated with linear accelerators with or without rotational Intensity Modulated RadioTherapy (IMRT).

**Methods:**

Patients with either hepatocellular carcinoma, cholangiocarcinoma or metastatic liver lesions who had one to three lesions treated with SBRT in a single institution were retrospectively reviewed. Tumor response was evaluated according to EASL criteria 3 months after SBRT completion using MRI and/or abdominal CT scan. Responses were categorised as: Stable Disease (SD), Partial Response (PR), Complete Response (CR), Local Progression or Distant Progression in cases of new intra-hepatic lesions out-of-field or extra-hepatic metastases. Local Control (LC), Progression Free Survival (PFS), Overall Survival (OS) and treatment-related toxicities are reported.

**Results:**

Between 2007 and 2012, 20 patients with a total of 24 lesions were treated with SBRT. Fourteen patients presented hepatocellular carcinoma (HCC), the others had either metastatic lesions from colorectal cancer (CRC) or cholangiocarcinoma. The median diameter of the lesions was 23 mm (5–98).

The dose per fraction ranged from 6 to 20 Gy with a median total dose of 60 Gy (range: 36–60 Gy). The dose was prescribed to the 80% isodose line covering the PTV.

The median follow-up was 24 months (15.7-29.7).

The actuarial LC rate was 78% for patients with HCC and 83% for those with adenocarcinoma and cholangiocarcinoma. Median OS was 37 months and OS rates were 83% at 12 and 24 months for HCC and 100% for adenocarcinoma. PFS was 54% for HCC and 50% for other types of tumors at 24 months.

Acute grade 3–4 toxicities occurred in 2 patients; a small proportion of the other patients experienced grade 1 or 2 toxicities.

**Conclusions:**

SBRT provides excellent local control with minimal side effects in selected patients.

## Background

HCC is the fourth most common cancer in the world and SBRT offers an interesting alternative to invasive management. Even though surgical resection remains the gold standard in the management of primary or metastatic liver disease, SBRT appears to be a treatment option for selected patients who are not eligible for surgery or invasive procedures [1,2]. Invasive procedures can provide comparable rates of long-term overall survival (OS), but pre-existing hepatic dysfunction, lesion size or tumor site can significantly limit these modalities with regard to patient eligibility and treatment side effects.

Besides, as the liver is a common site of metastases of many tumor types, patients with inoperable metastases should be considered for SBRT in order to improve local control, time to progression and OS. In particular, SBRT may be appropriate for selected patients suffering from « oligo-metastatic » disease while a few high dose fractions can provide local control rates higher than 70 to 80%, which may improve survival and quality of life [3-5].

The aim of this retrospective study was to evaluate the feasibility, features, tolerance and preliminary results obtained in a non-selected series of patients treated with SBRT delivered with or without rotational IMRT.

## Methods

Between March 2007 and August 2012, a total of 24 lesions in 20 consecutive patients were treated with SBRT and reviewed after approval from the Institutional review Board of the Georges François Leclerc Cancer Center. Informed consent (written or oral) was obtained from all patients in accordance with national laws. Fourteen patients suffered from HCC, four from liver metastases from colorectal cancer and two patients from cholangiocarcinoma. Adult patients with one to three hepatic lesions were eligible and included (Table 1). In most cases, the diagnosis of HCC was based on characteristic images on Magnetic Resonance Imaging (MRI) and high levels of alpha-foeto protein. Pathological confirmation was not required as long as established radiographic criteria were satisfied. These patients often had a previous but stable history of cirrhosis (11/14 HCC patients). All of them were Child-Turcotte-Plugh A during the treatment. All patients treated for metastatic lesions were evaluated with both MRI and PET-TDM. The patients’ characteristics are presented in Table 2.

● SBRT Technique

1. Acquisition

Patients were immobilized during CT simulation using a customized, external vacuum-type synthetic body mold from the neck to the pelvis. To account for tumor motion during the respiratory cycle, a Real-time Position Management (RPM, Varian Medical Systems, Palo Alto, CA) technique was chosen. As the patient is scanned, the respiration signal is simultaneously recorded. Once the images are acquired, they are post-processed into individual 3D image sets according to the respiratory phases. Patients were prepared to maintain regular breathing using audio breathing-training techniques.

2. Target definition

Because of the fast tumor wash-out of HCC, the contrast information provided by 4D CT scan is not accurate enough to delineate the target. We therefore fused CT images with recent MRI by using a rigid fusion procedure with four corresponding points on liver anatomical structures. Fiducials [2,3] already placed around the target contributed to the accuracy of the fusion process. The Gross Target Volume (GTV) was delineated on a contrast-enhanced treatment planning computed-tomography (CT) scan. It included the tumor in all phases of the normal respiratory cycle without respiratory gating or the tumor-tracking system. To delineate the Internal Target Volume (ITV), the GTV was expanded manually using data provided by the 4D CT scan showing fiducials motion and tumor position in different phases of the respiratory cycle. A median margin of 5 mm was added to the ITV to obtain the Planning Target Volume (PTV).

3. Dosimetry planning, prescription dose and treatment delivery

SBRT was planned and administered using dynamic conformal arcs or multiple fixed coplanar or non-coplanar fields generated by a linear-accelerator with energies of 6 to 18 MV. The choice of the technique has been made according to the tumor location. Considering stereotactic approach the dose prescription used the 80% isodose prescription line currently proposed in this setting. The dose per fraction and total dose were determined using the Dose Volume Histogram (DVH) and Organ At Risk (OAR) with a schedule of 36 to 60 Gy in 6 to 15 Gy per fraction. The normal liver was defined as the volume of liver not included in the PTV (Liver-PTV) and the dose-constraints protocol for normal liver specified that a minimum volume of 700 cc should receive a total dose less than 15 Gy. Daily image guidance using on-board cone-beam CT (CBCT) imaging was used to relocalize the target before each treatment delivery. No gating or tracking procedures have been used during treatment delivery.

● Follow-up and evaluation of response

● Acute toxicities were scored clinically once a week during treatment, then monthly and then three months after completion of the treatment. A clinical examination and a biological work up combined with a 3Tesla MRI or Abdominal CT scan were planned 3 months after radiation therapy and every subsequent 3 months for 2 years. Late toxicities were scored using the Common Terminology Criteria for Adverse Events scoring system (CTCAE v4.0).

● The response for each treated lesion was evaluated using EASL criteria. Complete response (CR) was defined as the absence of enhanced tumor area reflecting complete tissue necrosis. Partial response (PR) was a decrease greater than 50% of enhanced areas (partial tissue necrosis). Progressive disease (PD) was an increase greater than 25% in the size of at least one measurable irradiated lesion. Stable disease corresponded to a response between PD and PR.

● Study endpoints and statistics

● The primary endpoint was Local Control (LC). New or progressive lesions that developed within or at the margin of the PTV were scored as in-field local progression whereas lesions that developed outside the PTV were scored as distant progression inside or outside the liver. A lesion that developed in field but after 6 months of follow-up was termed local relapse. Secondary endpoints were toxicities, Progression-Free Survival (PFS) and Overall Survival (OS). Actuarial LC and OS curves were generated using the Kaplan-Meier method.

**Table 1:** Disease characteristics

**Baseline characteristics of disease**	
Total number of patients	20
Total number of lesions evaluated	24
Primary tumor: HCC	14 (70%)
Primary tumor: CRC	4 (20%)
Primary tumor: Cholangiocarcinoma	2 (10%)
Maximum lesion diameter and range	23,5 mm
	(12-98 mm)
Patients with one liver lesion	13 (65%)
Patients with two liver lesions	5 (25%)
Patients with three liver lesions	2 (10%)

HCC = HepatoCellular Carcinoma, CRC = Colorectal Cancer.

**Table 2:** Characteristics of patients and treatments

**Sex**	**Male = 19**	
	**Female = 1**	
**Age (years)**	Median = 73	Range = 50-85
**Time between diagnosis and SBRT**	Median = 5 months	Range = 1.25-32.8
**Follow-up**	24 months	Range = 16-30
**Tumor diameter**	Median = 23 mm	Range = 5-98 mm
**Margins**	Median = 5 mm	Range = 0-10 mm
**Median PTV Dose**	Median = 60 Gy	Range = 36-60 Gy
**ITV Volume**	Median = 36 cc	Range = 5-456 cc
**PTV Volume**	Median = 95 cc	Range = 5-1059 cc
**D700cc Liver-PTV**	Mean = 14 Gy	Range = 1-56 Gy
**Dmean Liver-PTV**	Mean = 16 Gy	Range = 2-80 Gy
**PTV Volume/Liver-PTV**	Median = 0.06	Range = 0-1.64

## Results

**Figure 1:**
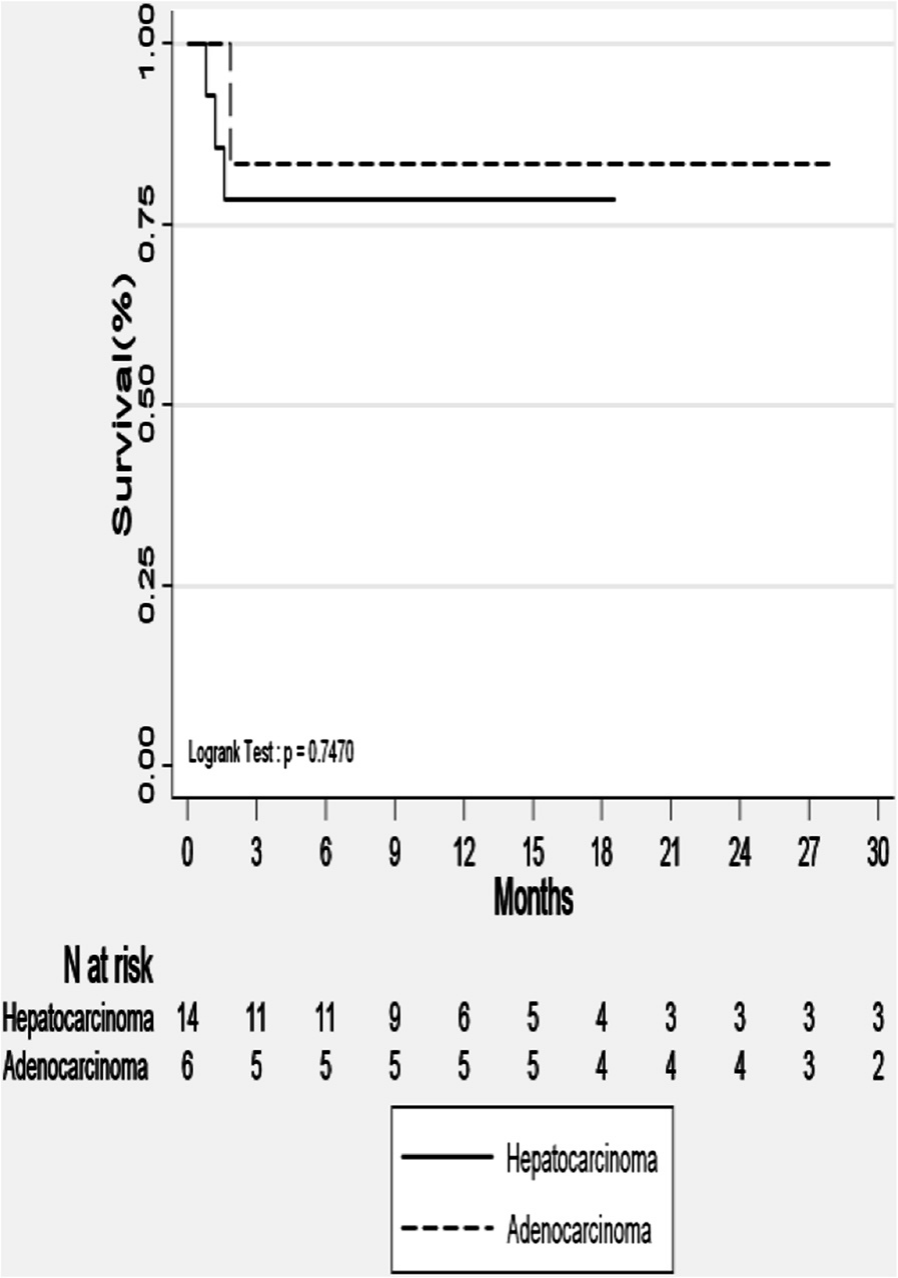
Local control.

20 consecutive patients with 24 liver lesions were treated with SBRT and reviewed. The median follow-up was 24 months. Fourteen lesions were treated with conventional conformal radiotherapy and 10 lesions with volumetric-modulated arctherapy. Among these 24 lesions, imaging studies showed a Complete Response (CR) in 10 patients, a Partial Response (PR) in 4 patients, and Stable Disease (SD) in 2 patients. The actuarial Local Control (LC) of the irradiated lesions at 12 months was 78% for HCC patients and 83% for the others (Figure 1).

**Figure 2:**
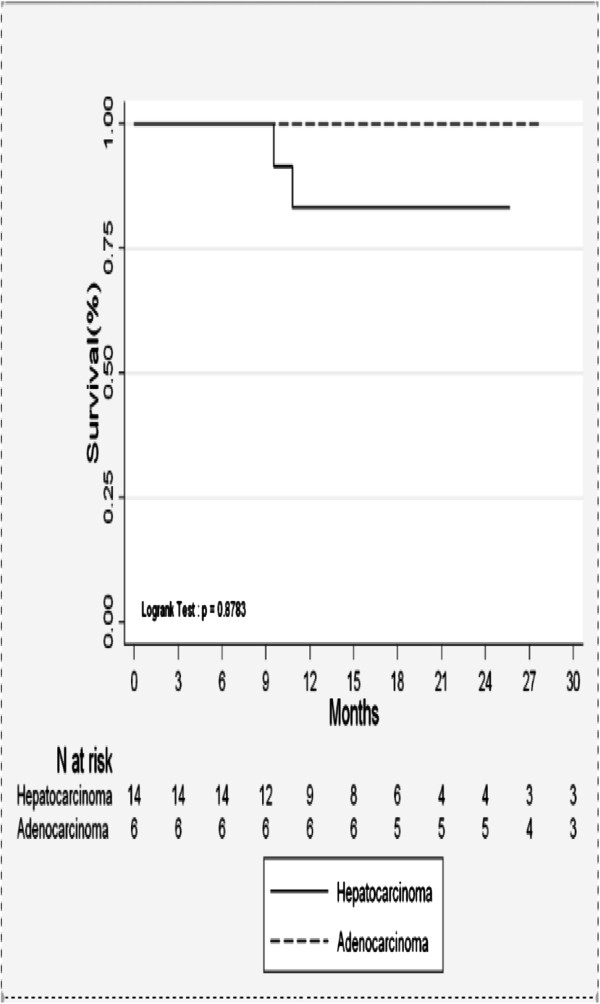
Overall survival.

**Figure 3:**
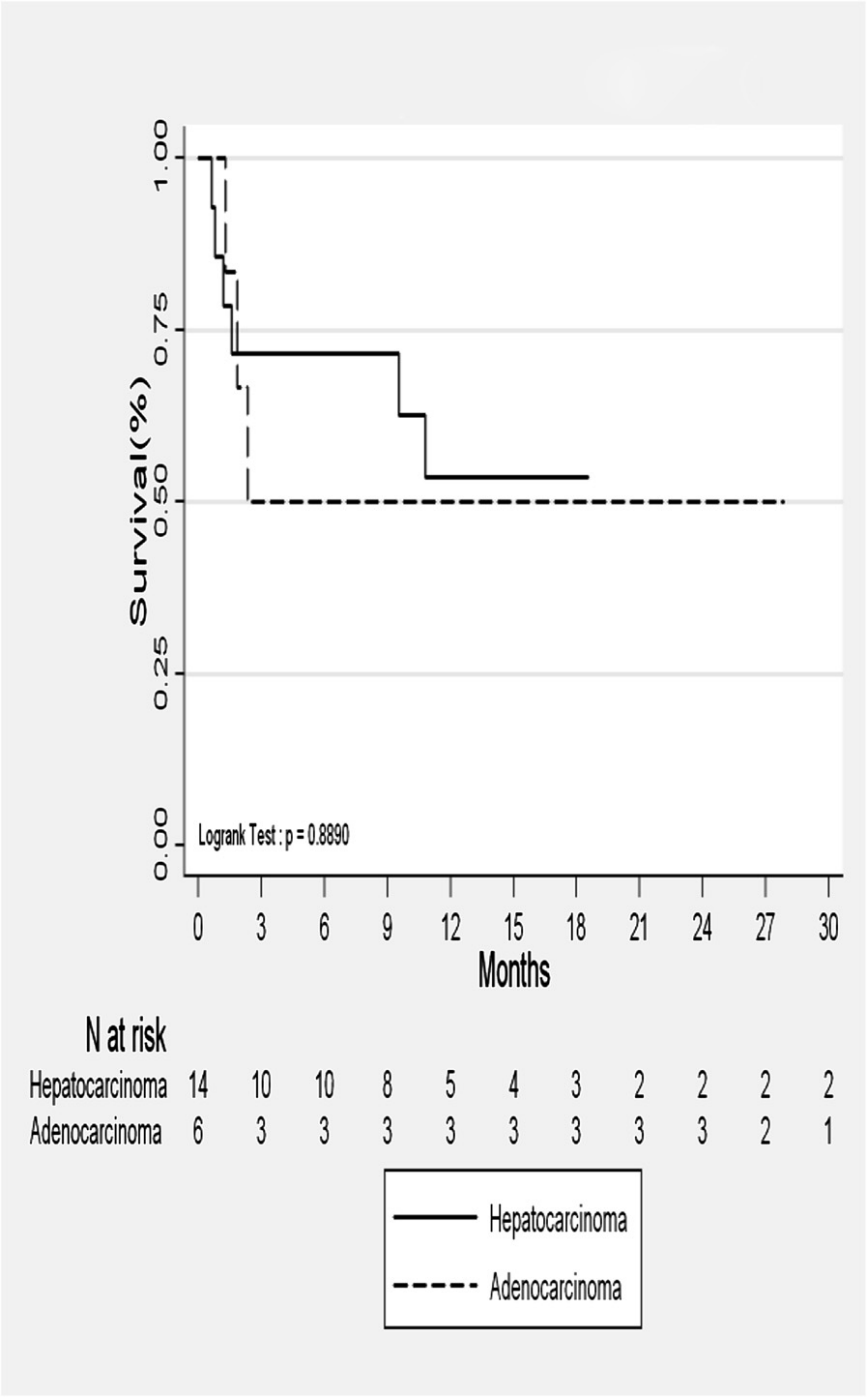
Progression free survival.

Of the responding patients, one subsequently developed in-field progression. This was recorded as recurrence or local relapse after CR. Four patients (21%) developed lesions in the liver but outside the radiotherapy field. Median OS was 37 months and actuarial OS by primary tumor site is shown in Figure 2. Progression-Free Survival (PFS) was 71% at 6 months and 53% at 24 months for HCC (Figure 3). For metastatic adenocarcinoma, it was 50% at 6 months (Figure 3).

### Post-SBRT treatment

Six patients with an initial partial response had, for local relapse or limited intra-hepatic progression 6 months after SBRT, chemo-embolization (three patients), radiofrequency ablation (RFA) (one patient), arterial chemotherapy (one patient) or surgery (one patient). Patients with distant progression received chemotherapy if possible or supportive care.

### Toxicities

The most common side effects were abdominal pain and nausea. Grade 1 or 2 toxicities occurred in 12 patients. Two patients experienced Grade 3 or more toxicity. The first one, with no history of cirrhosis, was treated for a large lesion (98 mm). Three months after completion of SBRT, he developed lethal Radiation Induced Liver Disease (RILD). D700cc was about 18 Gy. The second patient suffered from bleeding that required embolization. He was treated for a 6-cm diameter biopsy-proven HCC. D700cc was 11 Gy, D2cc to the stomach was 69.5 Gy. Initial complete response was recorded 6 months after completion of the radiotherapy. The patient died 8 months later from gastric bleeding. Both patients had been taking long-term bloodthinners for cardiovascular disorders. In our study, the median maximal dose delivered to the heart was 9.5 Gy without subsequent toxicity. There were no spinal cord or kidney toxicities. The maximum doses to the chest wall ranged from 15.8 Gy to 27.7 Gy, and there were no cases of pain or other complications such as rib fracture.

## Discussion

SBRT is emerging as a new non-invasive approach for local ablation in selected patients especially in patients with either liver-confined metastatic disease [6,7] or primary liver carcinoma [8]. Phase I investigations now suggest that SBRT can also be safely used to treat HCC in cirrhotic patients with Child-Turcotte-Plugh (CTP) classes A and B. Andolino et al. provided further support for the safety and efficacy of SBRT for HCC for patients with a CTP score ≤ 7 in a curative intent or as a bridge to transplant [1]. Some studies have confirmed the efficacy and safety of SBRT for metastatic lesions in an otherwise healthy liver [9,10].

In the definition of SBRT proposed by Timmerman, secure immobilization was the first point to ensure optimal treatment delivery [3]. This require adapted devices [11]. In Case’s study, during free-breathing, the average amplitude of liver motion was 1.4 mm in the medial-lateral plane, 9.0 mm in the cranial-caudal plane and 5.1 mm in antero-posterior plane [12]. Inter and intra-fraction variability in liver position in non-breath-hold SBRT lead to geometric uncertainties of no greater than 1 centimeter in the cranial-caudal plane and 0.5 cm in the axial plane. In our study, the ITV was recorded during the breathing cycle and the accuracy of the repositioning was ensured using CBCT before each fraction was delivered. Nonetheless, a 5 mm margin was applied around the ITV to create the PTV according to the pre-treatment evaluation of tumor motion on the 4D CT-scan.

RapidArc (RA) is a Volumetric Modulated Arc Therapy (VMAT) technique based on simultaneous optimisation of Multi Leaf Collimator (MLC) shapes, dose rate and gantry rotation speed. While the treatment dose is optimized and calculated on a static CT image, the motion of the target and the MLC may result in the delivered dose deviating from the planned dose.

Some authors have highlighted this issue and the dosimetric impact of leaf interplay with breathing-induced tumor motion. The reported differences were not significant when RA was delivered with two different arcs and within a single fraction plan [13]. One study concluded that the interplay between the motion of both the leaves and the target might induce an error in the delivered dose [14]. In our experience, in order to avoid such uncertainties in dose distribution, RA was not indicated for tumors located in the interface between the liver and the lung.

After the early experience of the University of Heidelberg of single-fraction SBRT for liver metastases updated in 2005, they reported 18-month LC rate of 66% following 22 Gy in one fraction [10]. On the basis of the relationship between LC and survival in surgical series and the observed dose–response with SBRT, Rusthoven et al. initiated a phase I/II multi-institutional trial of SBRT for liver metastases to evaluate the safety and efficacy of high dose SBRT [9]. Patients with one to three lesions were included and treated with a total dose ranging from 36 to 60 Gy in a phase I dose-escalation trial. The phase II dose was 60 Gy in 3 fractions and they demonstrated the feasibility and efficacy of high-dose SBRT with a few high-grade toxicities and high rates of LC. In our study, 14 patients received a total dose of 60 Gy: 9 in 4 fractions and 5 in 3 fractions; toxicities were low-grade.

In the setting of HCC, Andolino et al. [1] compared their results (with a median total dose between 40 and 44 Gy) with those reported by Tse et al. (median dose: 36 Gy) [15]. The former reported a LC rate of 90% at 2 years, while the latter reported an LC of 65% at 1 year. The most likely explanation could be a higher median dose per fraction and a lower median tumor volume.

Emerging new technologies that allow partial volume irradiation have lead us to reconsider the treatment of liver tumors by irradiation. Sawrie et al. proposed a review of normal tissue tolerance and toxicity in SBRT for liver metastases and primary HCC [16]. Each regimen provided a constraint to roughly one third of normal liver tissue and across all studies, threshold doses ranged from 7 to 21 Gy. Studies by Mendez Romero et al. [17] and Shefter et al. [18] were done with a critical volume constraint of 700 cc of normal liver that should not receive more than 15 Gy, assuming that the liver volume was at least 2,000 cc. Our data are consistent with other published data on SBRT while using similar constraints to the normal liver, acute and late liver toxicities were minimal for most patients. However, pretreatment hepatic function and the CTP score remains relevant and validated prognostic factors of complications in HCC.

Dawson et al. published a risk analysis of the probability of RILD using the Lyman NTCP model [19]. Their data confirmed that the liver exhibits a large volume effect for RILD, suggesting that the mean dose to the liver may be useful for ranking radiation plans with twice-daily fractions of 2 Gy. The dose-volume constraints usually applied in the literature are summarized in Table 3.

**Table 3:** Summary of dose-volume constraints for the liver

**Reference**	**Dose-volume**	**Dose-volume**
	**Constraints reported**	**Constraints converted to V(Gy)**
Herfarth *et al.*[10]	12 Gy to 30%	V12 ≤ 30%
	7 Gy to 50%	V7 ≤ 50%
Shefter *et al.*[18]	700 cc < 15 Gy	V15 ≥ 700 cc
Kavanagh *et al.*[6]		
Hoyer *et al.*[7]	10 Gy total < 30%	V10 < 30%
Mendez Romero *et al.*[17]	D33 < 21 Gy	V < 21 ≤ 33%
	D50 < 15 Gy	V15 ≤ 50%

Toxicities associated with SBRT for liver metastases were minimal if conservative dose-volume constraints for neighboring critical structures are respected for the stomach and small intestine. Mendez Romero et al. reported the following constraint: 5 cc of stomach should receive less than 21 Gy [17]. Few series have reported dose constraints to the heart, and a maximal dose of 7 Gy should be applied.

In 2001, the European Association for the Study of the Liver (EASL) conference concluded that RECIST criteria are not optimal to assess the response of liver tumors to locoregional treatment [20]. Conversely, EASL recognized that treatment-induced modifications in tumors correlated well with alterations in contrast enhancement patterns. Non-enhanced areas on post-SBRT dynamic imaging reflect necrosis, whereas viable tumor remains visible as a contrast-enhanced area. Therefore EASL criteria were used to assess tumor response in the present series. This is consistent with a previous report by Forner and al: they applied RECIST and EASL criteria to two prospective cohorts that had been treated with transarterial chemoembolization or percutaneous ablation for HCC [21]. They concluded that RECIST missed all of the CR and underestimated partial response compared with EASL. Furthermore, they found no correlation between EASL and RECIST and if RECIST criteria were used, then the rate of initial objective response would have been around 0%. Thus, the established efficacy of the treatment would have been obscured.

In the most recent series of liver SBRT, EASL criteria were used to assess tumor response. Price et al. reported their experience of SBRT on 26 patients with HCC [22]. SBRT delivered 24 to 48 Gy. MRI was performed every 3 months after treatment. At 12 months, eighteen of the 26 patients (69%) had more than 50% of non-enhancement, among whom thirteen had 100% of non-enhancement. However, only four patients had a complete response according to RECIST at the same time point.

Peritumoral enhancement should not be regarded as tumor progression and needs to be interpreted with caution. In the present series, imaging at 6 and 9 months distinguishes between progression and benign inflammation lesions that were difficult to analyze at 3 months. A first imaging evaluation at 6 months could be recommended. Furthermore, patients assessed according to EASL criteria have longer overall survival, thus justifying the use of EASL guidelines in series reporting the clinical results of local liver therapies for HCC.

## Conclusion

An international survey on radiotherapy for liver metastases suggests that radiation oncologists will be seeing more referrals for liver radiotherapy [23].

For HCC, SBRT with IGRT alone or combined with other loco-regional treatment such as Trans Arterial Chemo-Embolization (TACE) may play a significant role in the treatment of unresectable HCC.

Prospective studies are now needed to compare regimens and to identify parameters in order to optimize patient selection.

## Abbreviations

HCC: Hepato cellular carcinoma; SBRT: Stereotactic body radiation therapy; IGRT: Image guided radiation therapy; IMRT: Intensity modulated radiation therapy; MRI: Magnetic resonance imaging; CT: Computed tomography; EASL: European association for the study of the liver; RECIST: Response evaluation criteria in solid ttmors; CRC: Colo-rectal cancer; CTP: Child turcotte pugh; RILD: Radiation induced liver disease; RA: RapidArc; VMAT: Volumetric modulated arc therapy; MLC: Multi leaf collimator; SD: Stable disease; PR: Partial response; CR: Complete response; LC: Local control; PFS: Progression free survival; OS: Overall survival; GTV: Gross tumor volume; CTV: Clinical target volume; ITV: Internal target volume; PTV: Planning target volume; DVH: Dose volume histogram; OAR: Organ at risk; CBCT: Cone beam computed tomography; RFA: Radiofrequency ablation.

## Competing interests

The authors declare that they have no competing interests.

## Authors’ contributions

Conception and design: EN, AC, MQ, MRT, JC, PM. Acquisition of Data: EN, GC, MQ, MRT, JC. Analysis and interpretation of data: AC, EN, GC, RG, PM. Drafting the manuscript or revising it critically for important intellectual content: EN, GC, LM, RG, JC, PM. All authors read and approved the final manuscript.
